# First-year results of the Global Influenza Hospital Surveillance Network: 2012–2013 Northern hemisphere influenza season

**DOI:** 10.1186/1471-2458-14-564

**Published:** 2014-06-05

**Authors:** Joan Puig-Barberà, Anita Tormos, Anna Sominina, Elena Burtseva, Odile Launay, Meral A Ciblak, Angels Natividad-Sancho, Amparo Buigues-Vila, Sergio Martínez-Úbeda, Cedric Mahé

**Affiliations:** 1 Fundación para el Fomento de la Investigación Sanitaria y Biomédica de la Comunidad Valenciana (FISABIO-Salud Pública), Valencia, Spain; 2 Research Institute of Influenza, St. Petersburg, Russian Federation; 3 D.I. Ivanovsky Institute of Virology, Moscow, Russian Federation; 4 INSERM, Reseau National d’Investigation Clinique en Vaccinologie (REIVAC), Paris, France; 5 Université Paris Descartes, Sorbonne Paris Cité and Assistance Publique Hôpitaux de Paris, Hôpital Cochin, Paris, France; 6 National Influenza Reference Laboratory, Istanbul, Turkey; 7 Sanofi Pasteur, Lyon, France

**Keywords:** Influenza epidemiology, Surveillance network, Hospital

## Abstract

**Background:**

The Global Influenza Hospital Surveillance Network (GIHSN) was developed to improve understanding of severe influenza infection, as represented by hospitalized cases. The GIHSN is composed of coordinating sites, mainly affiliated with health authorities, each of which supervises and compiles data from one to seven hospitals. This report describes the distribution of influenza viruses A(H1N1), A(H3N2), B/Victoria, and B/Yamagata resulting in hospitalization during 2012–2013, the network’s first year.

**Methods:**

In 2012–2013, the GIHSN included 21 hospitals (five in Spain, five in France, four in the Russian Federation, and seven in Turkey). All hospitals used a reference protocol and core questionnaire to collect data, and data were consolidated at five coordinating sites. Influenza infection was confirmed by reverse-transcription polymerase chain reaction. Hospitalized patients admitted within 7 days of onset of influenza-like illness were included in the analysis.

**Results:**

Of 5034 patients included with polymerase chain reaction results, 1545 (30.7%) were positive for influenza. Influenza A(H1N1), A(H3N2), and both B lineages co-circulated, although distributions varied greatly between coordinating sites and over time. All age groups were affected. A(H1N1) was the most common influenza strain isolated among hospitalized adults 18–64 years of age at four of five coordinating sites, whereas A(H3N2) and B viruses were isolated more often than A(H1N1) in adults ≥65 years of age at all five coordinating sites. A total of 16 deaths and 20 intensive care unit admissions were recorded among patients with influenza.

**Conclusions:**

Influenza strains resulting in hospitalization varied greatly between coordinating sites and over time. These first-year results of the GIHSN are relevant, useful, and timely. Due to its broad regional representativeness and sustainable framework, this growing network should contribute substantially to understanding the epidemiology of influenza, particularly for more severe disease.

## Background

According to the World Health Organization (WHO), seasonal influenza epidemics affect an estimated 5–15% of the total population worldwide, with 3–5 million cases of severe illness, resulting in 250,000–500,000 deaths [1]. However, few data are available for many parts of the world where active surveillance is lacking. In addition, the viruses and the severity of influenza epidemics vary greatly between years and geographical areas [2-4]. To address the rapidly evolving antigenicity of circulating influenza viruses, twice annually, the WHO re-evaluates the viruses that should be included in the seasonal influenza vaccines. To inform policy decisions, national health authorities need to understand the burden of influenza disease and the impact of current vaccination programs in their countries.

High-quality, active surveillance networks are needed to better understand influenza epidemiology and therefore better control influenza epidemics [5-7]. Data from existing sentinel physician networks are used in several countries to conduct annual studies on the effectiveness of vaccines in preventing medically attended influenza-like illness (ILI) [8-12]. These networks, however, do not collect data on the impact of influenza infection on hospitalization or on the impact of influenza vaccines on influenza-related hospitalization, which substantially influence evaluation of the benefits and cost-effectiveness of influenza vaccines [13].

Active surveillance networks are also powerful advocacy instruments for highlighting the often-underestimated impact of influenza [5]. While hospital surveillance systems already exist for detecting outbreaks of respiratory viruses [14-16], few focus on the actual burden of serious influenza cases using the specific outcome of laboratory-confirmed influenza; instead, the burden is most often estimated from hospital databases using criteria prone to various biases [13,17].

The Global Influenza Hospital Surveillance Network (GIHSN) was initiated in 2011 to fill this gap in epidemiology and public health knowledge. The GIHSN is a public-private partnership between Sanofi Pasteur, FISABIO-Salud Pública, and several coordinating sites affiliated with national health authorities. In accordance with WHO recommendations [7], coordinating sites are selected based on their motivation, geographic representativeness, ability to conduct epidemiological studies, availability of laboratory facilities, and experience in influenza surveillance. Each coordinating site supervises a group of one to seven hospitals in its country or geographical region and follows a core reference protocol. The GIHSN has three main objectives: (i) evaluate the burden of severe influenza disease, defined as hospitalization related to community-acquired influenza or complications following an influenza infection; (ii) quantify the distribution of the different influenza viruses (A(H1N1), A(H3N2), B/Yamagata, and B/Victoria) among these severe cases; and (iii) measure the effectiveness of influenza seasonal vaccines to prevent these hospitalizations using a test-negative design.

In this report, we evaluated the characteristics of hospitalizations related to influenza and the temporal and geographic distribution of the different influenza viruses in these cases during the 2012–2013 Northern hemisphere influenza season, the program’s first year.

## Methods

### Study design

This was a multi-centre, prospective, active surveillance, hospital-based epidemiological study during the 2012–2013 influenza season in 21 hospitals in Spain, France, Turkey, and the Russian Federation (Table 1). Data on hospitalized patients with a diagnosis possibly associated with influenza were collected by an active surveillance system composed of healthcare professionals trained to follow a common reference protocol. Data were consolidated at five coordinating sites, including one in Spain (Valencia), one in France, one in Turkey, and two in the Russian Federation (Moscow and St. Petersburg).

**Table 1:** Hospital characteristics

**Country**	**Participating hospital**	**Type of Hospital**	**Total no. of beds**	**No. of beds for general medicine**	**No. of beds for paediatrics**	**No. of beds for geriatrics**	**No. of ICU beds**
Spain							
	General CS	General	580	437	79	0	58
	La Plana	General	251	100	19	0	9
	Pesset	General	540	211	41	0	16
	San Juan	General	350	230	37	0	13
	Elda	General	514	410	30	0	13
France							
	Cochin	-	1074	-	-	-	-
	Bichat	University hospital	1000	954	0	20	24
	Limoges	-	858	-	-	-	-
	Montpellier	-	3000	-	-	-	-
	Lyon	University hospital/tertiary care	683	603	0	64	53
Moscow							
	Hospital #1	Infectious diseases	1000	506	231	0	12
St. Petersburg							
	Hospital #30	Infectious diseases	1200	1175	0	-	25
	Hospital #5	Children's infectious diseases	650	0	635	0	15
	Hospital #4	Children's city hospital	360	0	345	0	15
Turkey							
	Hacettepe Univ. Hospital	-	1200	156	228	-	60
	Gazi Univ. Hospital	-	1150	-	-	-	-
	Trakya Univ. Hospital	University hospital	1042	951	72	0	50
	Istanbul Univ. Cerrahpaşa Hospital	University hospital	-	36	60	0	30
	Uludağ Univ. Hospital	-	1000	800	112	0	87
	Dr. Siyami Ersek Hospital	-	-	-	-	-	-
	Kartal Research Hospital	Research and education Hospital	880	750	90	0	41

Dashes indicate missing information. No., number. ICU, intensive care unit.

The reference protocol was adapted by the coordinating sites according to their local conditions. In particular, case identification was adapted to the specific local setting because of the differences in health care delivery systems between the different countries and differences in the types of hospitals included, although admission diagnosis codes for inclusion were the same for all sites. At each site, except Turkey, the study start was predefined according to the site experience of the influenza epidemic wave [8]. No study period was defined for Turkey because it was included as a pilot study. Specificities between coordinating sites in the application of the reference protocol are summarized in Table 2.

**Table 2:** Protocol specificities between coordinating sites

**Characteristic**	**Valencia, Spain**	**St. Petersburg, Russia**	**Moscow, Russia**	**Turkey**	**France**
**Hospitals**	5 health care district general hospitals	1 infectious disease hospital, 1 children’s city hospital, 1 children’s infectious disease hospital	1 infectious disease hospital	7 general hospitals	5 university hospitals
**Screening ward**	Emergency	Emergency and 3 selected wards	Paediatric, mixed, pregnancy, adults	Emergency and 7 selected wards	Emergency and >20 selected wards
**Screening diagnosis**	Admission diagnosis associated with influenza infection; clinical symptoms of ILI	Clinical symptoms of ILI	Admission diagnosis associated with influenza inflection; clinical symptoms of ILI	Clinical symptoms of ILI	Signs of respiratory illness for <7 days
**Recruited by**	Full-time trained nurses	Doctors	Doctors, nurses, heads of wards	Doctors	Doctors, clinical researchers, nurses
**Additional exclusion criteria**	Outside catchment area	-	-	-	Not affiliated with social security
**Study start criteria**	2 consecutive weeks with ≥2 cases	Week with ≥5 laboratory-confirmed influenza cases using national surveillance data	Week with ≥5 laboratory-confirmed influenza cases using national surveillance data	Not defined^a^	Defined by the national surveillance system
**Study end criteria**	2 consecutive weeks with no cases	Week with no laboratory-confirmed influenza cases	Week with no laboratory-confirmed influenza cases	Not defined^a^	Defined by the national surveillance system
**Actual study period (epidemiological weeks)**	2013-01 – 2013-15	2013-03 – 2013-22	2013-02 – 2013-21	2013-01 – 2013-14	2012-51 – 2013-16

^a^Study period not defined because it was a pilot study. Cases of ILI were reported between epidemiological weeks 1 and 14 of 2013.

Dashes indicate no additional exclusion criteria.

The GISHN study was approved by the local research ethics committees for each institution: Comité Ético de la Dirección General de Salud Pública y Centro Superior de Investigación en Salud Pública (CEIC-DGSP-CSISP), Spain; Comité de Protection des Personnes Ile-de-France III, France; Ethic Committee of Hospital #1 for Infectious Diseases of Moscow Health Department, the Russian Federation; Ethics Committee of the Research Institute of Influenza, St. Petersburg, the Russian Federation; Istanbul University, Istanbul Faculty of Medicine, Ethical Committee for Clinical Research, Turkey. All subjects included in the study or their legal representatives provided written informed consent following local research ethics boards’ requirements.

### Study population

Non-institutionalized patients hospitalized for at least 24 h with a diagnosis possibly associated with influenza were considered eligible to be included in the study (see Additional file 1: Table S1 for admission diagnosis codes). Patients in the Russian Federation, Spain, and Turkey could be of any age, whereas in France, only patients ≥18 years of age were screened.

Subjects ≥5 years of age had to meet the European Centre for Disease Prevention and Control’s clinical case definition of influenza-like-illness (ILI) [18], which included at least one of four systemic symptoms (fever or feverishness, headache, myalgia, or malaise) and at least one of three respiratory symptoms (cough, sore throat or shortness of breath), although we did not include sudden onset of symptoms as a criterion. Subjects ≥5 years of age also had to have been hospitalized within 7 days of the onset of ILI. Children <5 years of age had to have been admitted to the hospital within 7 days of the appearance of symptoms potentially associated with influenza. Patients were excluded if they had been discharged from a hospital within 30 days of the current admission.

### Data and sample collection

A nasopharyngeal swab was obtained from all patients. An additional pharyngeal swab was collected for patients ≥14 years of age and a nasal sample for children <14 years old. Samples were collected within 48 h of hospital admission and were stored at -20°C at the study site or were sent directly to the coordinating site’s laboratory for testing. Influenza infection status, patient demographics, and influenza vaccination status were recorded with a core questionnaire via a combination of face-to-face interview of patients or legal representatives, interviews of attending physicians, and a review of clinical records. Whenever possible, information about vaccination status collected with the core questionnaire was validated by existing registers or vaccination cards or by contacting the place where patients were administered the vaccine. A patient was considered as having received the current season’s influenza vaccination if their records demonstrated or if they recalled receiving it >14 days before the onset of ILI.

### Confirmation of influenza infection

RNA extraction and multiplex reverse transcriptase-polymerase chain reaction (RT-PCR) were performed at the coordinating sites to detect influenza A (H3N2), A (H1N1)pdm09, B/Yamagata, and B/Victoria according to local laboratory procedures. Coordinating sites in France, the Russian Federation, and Turkey are WHO National Influenza Centres, and the coordinating site in Valencia, Spain used the WHO RT-PCR protocol. Details of the RT-PCR methods are provided in the Additional file 2: Supplemental methods.

### Data management and statistical analysis

Coordinating sites collected anonymized data from the core questionnaires and checked for missing, inconsistent, or incorrect data. Whenever possible, queries of any inconsistencies or missing data were resolved by the investigators at each of the coordinating sites. Missing data were not replaced for the statistical analyses. Data from each coordinating site were shared with the network coordinating centre (FISABIO-Salud Pública, Valencia, Spain) via a secured, internet-based system.

Only samples taken within 7 days of symptom onset were included in the analysis. Descriptive statistics were calculated for the characteristics of influenza-associated hospitalization by age group, influenza virus and socio-demographic characteristics.

Statistical analysis and data management was performed using STATA version 12 (StataCorp, College Station, TX).

## Results

A total of 8795 patients hospitalized for diagnoses possibly related to influenza were considered eligible for this study (Table 3). Informed consent was obtained from 8162 (92.8%), who were subsequently screened for inclusion. A total of 5034 patients with valid RT-PCR results were included and, of these, 1545 (30.7%) were positive for influenza. Overall, the largest contributors of influenza-positive samples were St. Petersburg (n = 652; 42.2%) and Moscow (n = 471; 30.5%), followed by Spain (n = 236; 15.3%) and France (n = 150; 9.7%). Turkey, included as a pilot study, accounted for 2.3% (n = 36) of the laboratory-confirmed influenza cases. Hospitalization with confirmed influenza was recorded for 15.7% of the included subjects for Spain, 33.8% for Moscow, 39.6% for St. Petersburg, 34.7% for France, and 65.5% for Turkey.

**Table 3:** Patient disposition, samples processed, and number of admissions with laboratory-confirmed influenza by coordinating site

**Characteristic**	**Valencia, Spain**	**St. Petersburg, Russia**	**Moscow, Russia**	**Turkey**	**France**	**Overall**
**Records received, n**	5038	1986	1677	67	447	9215
Institutionalized, n	329	7	21	0	0	357
Non-resident ^a^, n	63	-	-	-	-	63
**Eligible, n**	4646	1979	1656	67	447	8795
Unable to communicate with patient or proxy, n	250	47	2	1	0	300
Consent not given, n	156	166	11	0	0	333
**Screened for inclusion, n**	4240	1766	1643	66	447	8162
Did not meet inclusion/exclusion criteria, n	1872	106	128	11	12	2129
Swab not taken, n	5	7	115	0	0	127
**Eligible and tested, n**	2363	1653	1400	55	435	5906
RT-PCR results missing or invalid, n	67	2	8	0	2	79
**Samples processed with RT-PCR results, n**	2296	1651	1392	55	433	5827
Sample taken outside the defined influenza season, n	789	3	0	0	1	793
**Samples with valid RT-PCR results, n**	1507	1648	1392	55	432	5034
Influenza-positive, n (%)	236 (15.7)	652 (39.6)	471 (33.8)	36 (65.5)	150 (34.7)	1545 (30.7)

Dashes indicate category not applicable.

^a^Spain only.

### Epidemiology of hospitalization with laboratory-confirmed influenza

**Figure 1:**
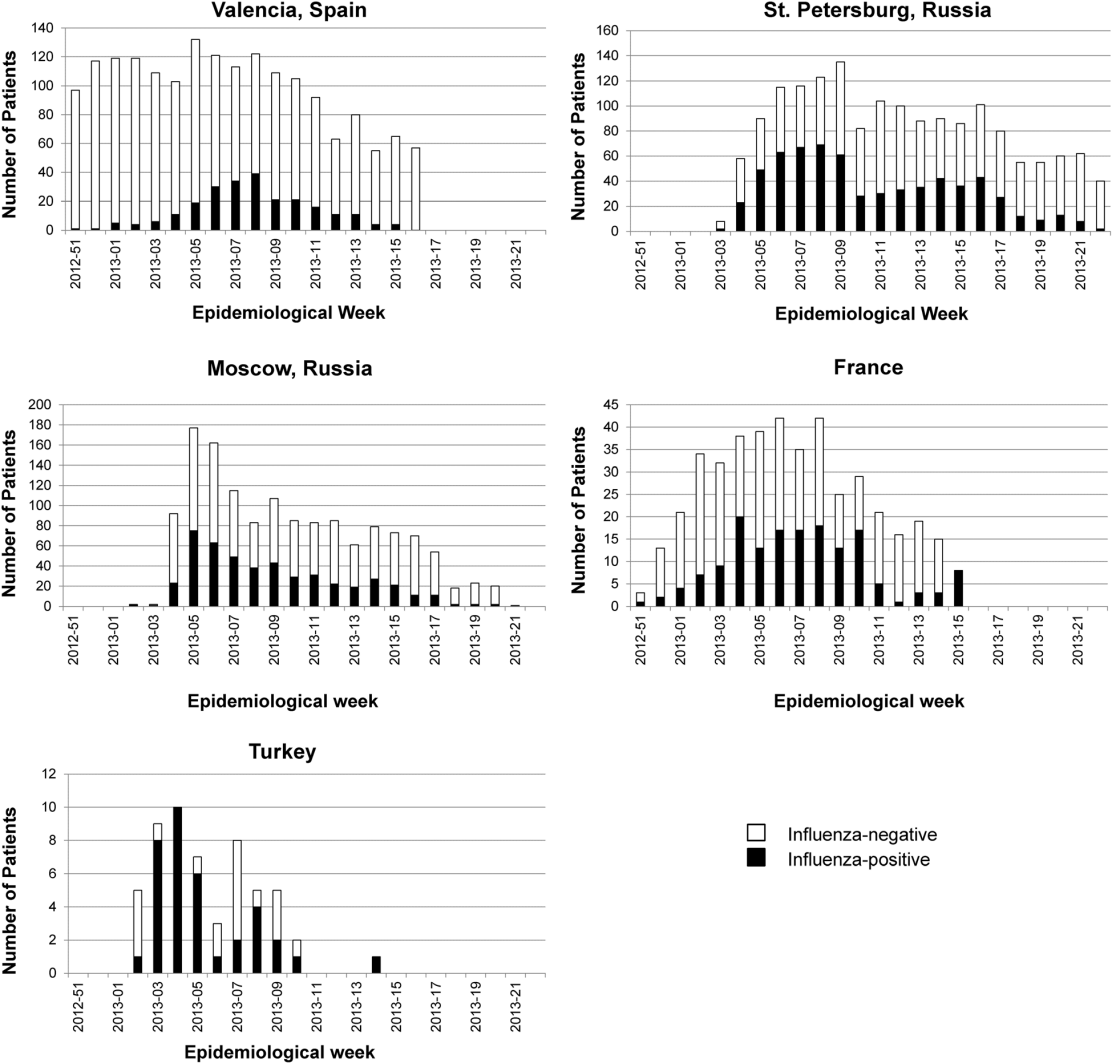
**Hospitalized influenza-positive and -negative cases by epidemiological week.** Shown are the number of ILI cases with valid RT-PCR samples and positive (laboratory-confirmed influenza; filled bars) or negative (open bars) for influenza virus.

**Figure 2:**
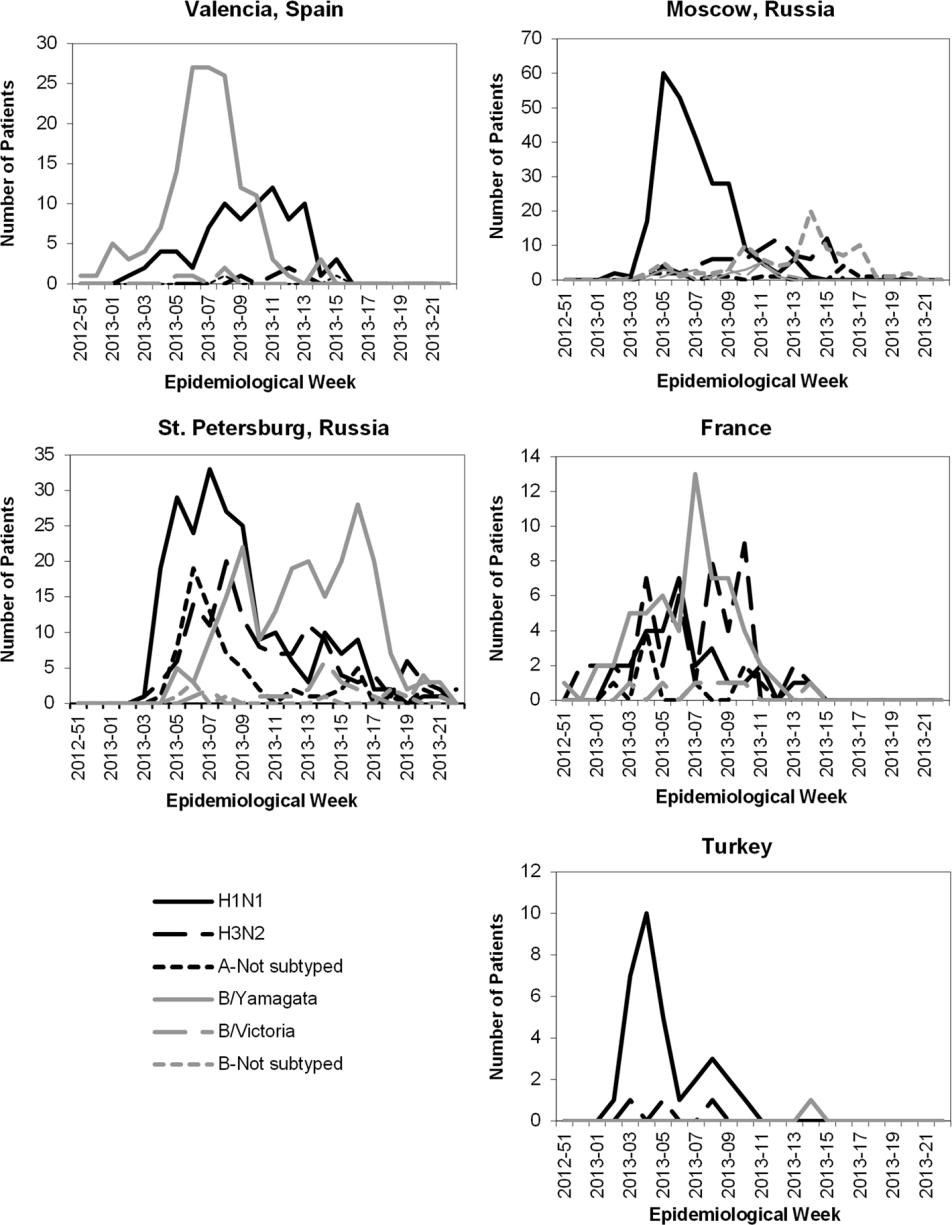
Admissions with influenza by virus and epidemiological week.

Cases hospitalized with confirmed influenza peaked between the fourth and eighth epidemiological week of 2013 (Figure 1). The timing of suspected influenza cases generally paralleled that of confirmed cases except in Spain, where the number remained elevated for the duration of the study because of the boarder criteria for case identification. For all countries, the influenza season, as defined by the appearance of confirmed influenza cases, started around the beginning of 2013 (epidemiological week 51 of 2012 to week 3 of 2013) and lasted until epidemiological week 14 to 22 of 2013. The influenza season was similar in Moscow and St. Petersburg, although in St. Petersburg, influenza cases remained elevated over a longer period with no clear peak. In Spain and France, reporting of influenza cases began and ended earlier.All four influenza viruses examined in this study (A(H1N1), A(H3N2), B/Yamagata, and B/Victoria) contributed to hospitalization with influenza at all five coordinating sites. The timing and distribution of viruses varied considerably between the coordinating sites (Figure 2). In Moscow, A(H1N1) was the most frequent cause of confirmed influenza (53.7% of cases), peaking at epidemiological week 5 of 2013, followed by an increase in B and A(H3N2) towards the end of the season. A(H1N1) was the most frequent cause of confirmed influenza in Turkey, although results for the full season were not available. In St. Petersburg, A(H1N1) was a frequent cause of confirmed influenza (33.4%), A(H3N2) (20.2%) was also common and B lineages (37.9% overall) appeared later. In Spain, B/Yamagata was the most frequent (60.6% overall) and peaked around epidemiological week 6 of 2013, A(H1N1) increased later in the season. In France, the frequency of B virus (44.0% overall) was slightly higher than of the other influenza viruses, although it co-circulated with the influenza A viruses. Both B virus lineages circulated in all countries. The lineage included in the vaccine (B/Yamagata) was most common, representing 89.2% (436/489) of all B viruses cases in which the lineage was identified.

At all coordinating sites except for France, A(H1N1) was the most common influenza strain isolated from hospitalized adults 18–64 years of age. In contrast, A(H3N2) and B viruses were more common than A(H1N1) in elderly adults (≥65 years of age) at all coordinating sites (Table 4).

**Table 4:** Influenza cases detected by coordinating site, virus, and age group

	**Valencia (N = 236)**			**St. Petersburg (N = 652)**			**Moscow (N = 471)**			**Turkey (N = 36)**			**France (N = 150)**	
**Virus**	**< 18 y**	**18-64 y**	**≥ 65 y**	**< 18 y**	**18-64 y**	**≥65 y**	**< 18 y**	**18-64 y**	**≥ 65 y**	**< 18 y**	**18-64 y**	**≥ 65 y**	**18-64 y**	**≥ 65 y**
**Total A**	20 (40.0)	29 (54.7)	40 (30.1)	240 (64.0)	171 (66.0)	11 (61.1)	51 (64.6)	284 (75.9)	10 (55.6)	9 (100.0)	17 (94.4)	9 (100.0)	43 (64.2)	43 (51.8)
A(H1N1)	19 (38.0)	28 (52.8)	35 (26.3)	113 (30.1)	100 (38.6)	5 (27.8)	30 (38.0)	217 (58.0)	6 (33.3)	9 (100.0)	16 (88.9)	7 (77.8)	20 (29.9)	10 (12.0)
A(H3N2)	0 (0.0)	1 (1.9)	4 (3.0)	78 (20.8)	48 (18.5)	6 (33.3)	18 (22.8)	53 (14.2)	4 (22.2)	0 (0.0)	1 (5.6)	2 (22.2)	18 (26.9)	29 (34.9)
A - Not subtyped	1 (2.0)	0 (0.0)	1 (0.8)	49 (13.1)	23 (8.8)	0 (0.0)	3 (3.8)	14 (3.7)	0 (0.0)	0 (0.0)	0 (0.0)	0 (0.0)	5 (7.5)	4 (4.8)
**Total B**	30 (60.0)	24 (45.3)	93 (69.9)	144 (38.4)	96 (37.1)	7 (38.9)	28 (35.4)	91 (24.3)	8 (44.4)	0 (0.0)	1 (5.6)	0 (0.0)	26 (38.8)	40 (48.2)
B/Yamagata	28 (56.0)	23 (43.4)	92 (69.2)	127 (33.9)	80 (30.9)	6 (33.3)	6 (7.6)	12 (3.2)	2 (11.1)	0 (0.0)	1 (5.6)	0 (0.0)	20 (29.9)	39 (47.0)
B/Victoria	2 (4.0)	1 (1.9)	1 (0.8)	10 (2.7)	14 (5.4)	1 (5.6)	2 (2.5)	15 (4.0)	0 (0.0)	0 (0.0)	0 (0.0)	0 (0.0)	6 (9.0)	1 (1.2)
B - Not subtyped	0 (0.0)	0 (0.0)	0 (0.0)	7 (1.9)	2 (0.8)	0 (0.0)	20 (25.3)	64 (17.1)	6 (33.3)	0 (0.0)	0 (0.0)	0 (0.0)	0 (0.0)	0 (0.0)
**Total**	50 (100)	53 (100)	133 (100)	375 (100)	259 (100)	18 (100)	79 (100)	374 (100)	18 (100)	9 (100)	18 (100)	9 (100)	67 (100)	83 (100)

Values are n (%). Patients may have had more than one influenza virus due to a mixed infection.

### Demographics and clinical features of hospitalized laboratory-confirmed influenza cases

In Spain, France, and Turkey, more than half of influenza cases had one or more comorbidities, whereas at both coordinating sites in the Russian Federation, more than 80% were reported to have no comorbidities (Table 5). Cardiovascular disease, respiratory disease, and diabetes were the most frequent comorbidities at all coordinating sites among positives for influenza.

**Table 5:** Characteristics of subjects hospitalized with laboratory-confirmed influenza

**Characteristic**	**Valencia, Spain**	**St. Petersburg, Russia**	**Moscow, Russia**	**Turkey**	**France**
**Age**	N = 236	N = 652	N = 471	N = 36	N = 150
<18 y	50 (21.2)	375 (57.5)	79 (16.8)	9 (25.0)	-
18 - 64 y	53 (22.5)	259 (39.7)	374 (79.4)	18 (50.0)	67 (44.7)
** ≥**65 y	133 (56.4)	18 (2.8)	18 (3.8)	9 (25.0)	83 (55.3)
**Sex**	N = 236	N = 652	N = 471	N = 36	N = 150
Male	124 (52.5)	347 (53.2)	151 (32.1)	22 (61.1)	69 (46.0)
Female	112 (47.5)	305 (46.8)	320 (67.9)	14 (38.9)	81 (54.0)
Pregnant^a^	1 (0.9)	1 (0.3)	242 (75.6)	1 (7.1)	4 (4.9)
**Number of comorbidities**	N = 236	N = 652	N = 471	N = 36	N = 150
0	97 (41.1)	587 (90.0)	383 (81.3)	8 (22.2)	36 (24.0)
1	69 (29.2)	46 (7.1)	70 (14.9)	14 (38.9)	52 (34.7)
≥2	70 (29.7)	19 (2.9)	16 (3.4)	14 (38.9)	62 (41.3)
**Chronic conditions**	N = 236	N = 652	N = 471	N = 36	N = 150
Cardiovascular disease	54 (22.9)	48 (7.4)	40 (8.5)	15 (41.7)	49 (32.7)
Respiratory disease	77 (32.7)	17 (2.6)	23 (4.9)	10 (27.8)	61 (40.7)
Diabetes	56 (23.7)	12 (1.8)	4 (0.9)	7 (19.4)	33 (22.2)
Immunodeficiency	7 (3.0)	0 (0.0)	0 (0.0)	7 (19.4)	14 (9.3)
Renal impairment	18 (7.6)	1 (0.2)	18 (3.8)	5 (13.9)	18 (12.0)
Rheumatologic disease	4 (1.7)	0 (0.0)	0 (0.0)	3 (8.3)	17 (11.3)
Neuromuscular disease	12 (5.1)	0 (0.0)	4 (0.9)	5 (13.9)	6 (4.0)
Cirrhosis	9 (3.8)	3 (0.5)	10 (2.1)	2 (5.6)	1 (0.7)
Neoplasm	5 (2.1)	4 (0.6)	6 (1.3)	8 (22.2)	18 (12.0)
**Vaccinated 2012-2013**	N = 236	N = 652	N = 471	N = 36	N = 150
Yes	82 (34.8)	11 (1.7)	6 (1.3)	5 (13.9)	50 (33.3)
No	154 (65.3)	641 (98.3)	465 (98.7)	31 (86.1)	100 (66.7)
**Outcome**	N = 236	N = 652	N = 471	N = 36	N = 150
ICU admission	4 (1.7)	3 (0.5)	2 (0.4)	11 (30.6)	-
Death	5 (2.1)	0 (0.0)	0 (0.0)	7 (19.4)	4 (2.7)

All values are number of subjects with percent in brackets. No data were missing. -, not reported.

^a^Calculated relative to females.

In Spain and France, more than half of the subjects with influenza were ≥65 years of age, whereas <5% in St. Petersburg and Moscow were in this age group. Most of the subjects with influenza in Moscow were 18–64 years of age, while in St. Petersburg, the only site including dedicated paediatric hospitals, most were <18 years of age. The male-to-female ratio for confirmed influenza cases was close to 1 in Spain, St. Petersburg, and France, whereas in Moscow, more women than men were included. Indeed, a maternity ward was included in this site, where 242 pregnant women had influenza. Amongst women 15 to 45 years of age hospitalized for diagnoses possibly related to influenza, pregnancy was significantly associated with a risk of confirmed influenza (adjusted OR, 1.36 [95% CI, 1.03 – 1.80]).

A total of 16 deaths and 20 intensive care unit admissions were recorded. Outcomes were similar at the different coordinating sites, except for Turkey, which had a disproportionately high number of serious outcomes (11 intensive care unit admissions and 7 deaths). For the 16 deaths, the mean age was 60 years (range, 1 – 93 years). Nine of these patients were >65 years of age, two had been vaccinated against seasonal influenza during the current season, and seven had more than one comorbidity.

At all five coordinating sites, the length of hospital stay was similar for all influenza circulating viruses (Additional file 3: Figure S1).

### Influenza vaccine uptake

Influenza vaccination coverage among all hospitalized patients included in the study, whether positive or negative for influenza, was low in St. Petersburg (1.4%), Moscow (1.7%), and Turkey (14.5%) and moderate in Spain (38.9%) and France (47.0%). Influenza vaccination among all patients was highest in elderly adults in Spain (60.7%), Turkey (23.1%), and France (63.8%). In hospitalized children, regardless RT-PCR results, uptake rates at all coordinating sites were very low (≤5%).

## Discussion

These results represent the first year’s experience from the GIHSN, a prospective hospital-based influenza surveillance network. The data presented are from the 2012–2013 Northern hemisphere influenza season and were collected from 21 hospitals via five coordinating sites in four countries. Of a total of 6033 eligible patients, valid samples were obtained from 5034, of whom 1545 (30.7%) were positive for influenza.

The study confirmed that influenza can result in serious outcomes not only in elderly people and those with comorbidities but also in the wider population, irrespective of age or sex. It also confirmed that pregnant women are at significant risk for hospitalization with influenza [19,20], which supports global recommendations for prioritizing influenza vaccination for pregnant women [21,22].

The study allowed us to examine the influenza viruses resulting in hospitalization. We found substantial heterogeneity in virus circulation between coordinating sites, even those in neighbouring geographical areas and even within the same site over time. Unexpectedly, influenza B, mostly the Yamagata lineage, was the most common virus identified in elderly adults, where it accounted for more than half of the hospitalized confirmed influenza cases. Thus, as found in a recent structured review [23], influenza B poses a significant health burden. Indeed, influenza B is now receiving increased attention, and quadrivalent influenza vaccines containing both B lineages are now becoming available. Sample sizes were not large enough to examine influenza viral strains according to comorbidities and other risk factors including pregnancy, but this should become possible as the GIHSN grows and sample sizes increase.

Although the patients included in this study were not necessarily representative of the overall population (i.e. subset of hospitalized patients with a diagnosis possibly associated with influenza), vaccination rates in these patients were lower than recommended by the WHO for European countries [24]. The French patients in this study, regardless their RT-PCR result, had the highest vaccination rate at 47.0%, which was similar to the overall rate for France reported in 2010–2011 (50.4%) [25]. However, it is not unusual to observe some influenza cases in vaccinated individuals because seasonal influenza vaccines have only a moderate protecting effect, especially in elderly adults [26]. The lowest vaccination rates for these patients were in the Russian Federation, where fewer than 2% reported being vaccinated for seasonal influenza. This may have been due to the peculiarity of the groups enrolled (e.g. mostly pregnant women in Moscow and mostly children in St. Petersburg), poor uptake, the ability of the vaccine to prevent hospitalization, or a combination of these factors, although these influences remain to be assessed over the coming years.

The findings of this study are strengthened by the active surveillance methodology, specific definitions at each site of admission possibly related to an influenza infection, confirmation of influenza cases by RT-PCR, and the consistent body of evidence generated across coordinating sites. In addition, all RT-PCR was carried out by WHO National Influenza Centres (France, Moscow, St. Petersburg, and Turkey) or using the WHO protocol (Spain).

The results should be generally applicable because of the diversity of participating hospitals and healthcare settings. Nevertheless, the adaptation of case identification to the specific local setting, mainly driven by practical considerations, might have increased the sensitivity of the results to geographical variation. For future years, to help reduce heterogeneity between sites, we plan a common standard operating procedure and meetings at the beginning of the study to share pilot data and harmonise criteria and follow-up activities.

## Conclusion

These first-year findings demonstrate that the GIHSN produces relevant, important, and timely data. Despite the size of the network, results were available before the start of the following influenza season. These results will help understand and prepare for seasonal influenza epidemics. In particular, this platform provides annual data on the severe end of the influenza infection spectrum, as represented by hospitalized cases, for a wide range of populations. More formal burden and risk assessment will become available as documentation of the catchment population of these hospitals and wards improves. By adding the ability to evaluate the impact of vaccination programs, these data support those collected by SARI surveillance systems, although the GIHSN employs a broader definition of ILI that encompasses systemic symptoms beyond fever (i.e., malaise, headache, and myalgia).

The breadth of results provided by the GIHSN are particularly useful for assessing the effectiveness and cost-effectiveness of intervention programs, and they open the door to further research and public health activities, especially on risk factors and the impact of influenza and influenza vaccination programs in different populations. The approach used by the GIHSN also allows additional components to be added to respond to other scientific questions. During this first year of the GIHSN, only 21 hospitals and four countries were included, but additional hospitals and countries, including some from the Southern hemisphere, have already agreed to join the network. Thus, the GIHSN, with its broad regional representativeness and sustainable framework, should continue to contribute significantly to our knowledge of influenza epidemiology.

## Competing interests

JPB works for the Vaccines Research Area of FISABIO, which has received funding from GlaxoSmithKline, Novartis, Pfizer, Sanofi Pasteur, and Sanofi Pasteur-MSD for conducting studies on infectious disease epidemiology, vaccine effectiveness, pharmacoeconomics, and safety. The Vaccines Research Area is and has been involved in various randomized clinical trials with GlaxoSmithKline, Novartis, Pfizer, and MSD vaccines. AT is a member of a program funded by Sanofi Pasteur. OL has been an investigator on vaccine studies sponsored by and has received travel support to attend scientific meetings from Sanofi Pasteur and other pharmaceutical companies. CM is an employee of Sanofi-Pasteur. All other authors declare no conflicts of interest.

## Authors’ contributions

JPB participated in the design of the study; collected, analysed, and interpreted data; participated in preparing the article; and approved the final article. AT, ANS, and ABV participated in the design of the study; analysed and interpreted data; participated in preparing the article; and approved the final article. AS, EB, OL, and MAC participated in the design of the study; collected and interpreted data; participated in preparing the article; and approved the final article. SMU analysed and interpreted data; participated in preparing the article; and approved the final article. CM participated in the design of the study, interpretation of the data; writing of the manuscript; and approved the final article.

## Pre-publication history

The pre-publication history for this paper can be accessed here:

http://www.biomedcentral.com/1471-2458/14/564/prepub

## Supplementary Material

Additional file 1: Table S1 Admission diagnoses possibly associated with an influenza infection according to the International Classification of Diseases (ICD) versions 9 and 10. Click here for file

Additional file 2 Supplemental methods. Click here for file

Additional file 3: Figure S1 Length of hospital stay by coordinating site and influenza strain. Boxes indicate interquartile ranges, bars indicate the upper and lower adjacent values, and points indicate outliers. Click here for file
