# Supplementary material for: First-year results of the Global Influenza Hospital Surveillance Network: 2012–2013 Northern hemisphere influenza season
Source: BMC Public Health. 2014 Jun 5;14:564. doi: 10.1186/1471-2458-14-564 (PMC4057821; doi:10.1186/1471-2458-14-564)
Supplement: Additional file 1: Table S1 — Admission diagnoses possibly associated with an influenza infection according to the International Classification of Diseases (ICD) versions 9 and 10. [file 1471-2458-14-564-S1.doc]

Supplemental Table 1. Admission diagnoses possibly associated with an influenza infection according to the International Classification of Diseases (ICD) versions 9 and 10.

| **Patients ≥ 5 years of age** | **ICD 9 Codes** | **ICD 10 Codes** |
| --- | --- | --- |
| **Acute respiratory infection** | 382.9; 460-466 | J00-J06, J20-J22, H66.90 |
| **Acute myocardial infarction or acute coronary syndrome** | 410-411 and 413-414 | I20-I25.9 |
| **Asthma** | 493-493.92 | J45.2-J45.22, J45.9-J45.998, J44-J44.9 |
| **Heart failure** | 428-429.0 | I50-I50.9; I51.4 |
| **Pneumonia and influenza** | 480-488 | J09-J18 |
| **Chronic Pulmonary Obstructive disease** | 490, 491, 492, 496 | J40-J44.9 |
| **Myalgia** | 729.1 | M79.1 |
| **Metabolic failure (diabetic coma, renal dysfunction, acid-base disturbances, alterations to the water balance)** | 250.1- 250.3; 584-586; 276-277 | E11.9, E10.9, E11.65, E10.65, E10.11, E11.01, E10.641, E11.641, E10.69, E11.00, E10.10, E11.69, N17.0, N17.1, N17.2, N17.8, N17.9, N18.1, N18.2, N18.3, N18.4, N18.5, N18.6M N18.9, N19, E87.0, E87.1, E87.2, E87.3, E87.4, E87.5, E87.6, E87.70, E87.71, E87.79, E86.0, E86.1 |
| **Altered consciousness, convulsions, febrile-convulsions** | 780.01-780.02; 780.09; 780.31-780.32 | R40.20, R40.4, R40.0, R40.1, R56.00, R56.01 |
| **Dyspnoea/respiratory abnormality** | 786.0 | R06.0, R06-R06.9 |
| **Respiratory abnormality** | 786.00 | R06.9 |
| **Shortness of breath** | 786.05 | R06.02 |
| **Respiratory abnormality nec** | 786.09 | R06.3, R06.00, R06.09, R06.83 |
| **Respiratory symptoms/chest symptoms** | 786.9 | R06.89 |
| **Fever or fever unknown origin or non-specified** | 780.6-780.60 | R50, R50.9 |
| **Cough** | 786.2 | R05 |
| **Sepsis, Systemic inflammatory response syndrome** | 995.90-995.94 | R65.10, R65.11, R65.20, A41.9 |
| **Patients 0–4 years of age** | **ICD 9 Codes** | **ICD 10 Codes** |
| **Acute upper or lower respiratory disease** | 382.9; 460 to 466 | J00-J06, J20-J22 |
| **Dyspnoea, breathing anomaly, shortness of breath, tachypnea** | 786.0; 786.00; 786.05-786.07; 786.09; 786.9 | R06.0, R06, R06.9, R06.3, R06.00, R06.09, R06.83, R06.02, R06.82, R06.2, R06.89 |
| **Asthma** | 493-493.92 | J45.2-J45.22, J45.9-J45.998, J44-J44.9 |
| **Pneumonia and influenza** | 480 to 488 | J09-J18 |
| **Heart failure** | 428-429.0 | I50-I50.9; I51.4 |
| **Myalgia** | 729.1 | M79.1 |
| **Altered consciousness, convulsions, febrile convulsions** | 780.01-780.02; 780.09; 780.31-780.32 | R40.20, R40.4, R40.0, R40.1, R56.00, R56.01 |
| **Fever or fever unknown origin or non-specified** | 780.6-780.60 | R50, R50.9 |
| **Cough** | 786.2 | R05 |
| **Gastrointestinal manifestations** | 009.0; 009.3 | A09.0; A09.9 |
| **Sepsis, Systemic inflammatory response syndrome** | 995.90-995.94 | R65.10, R65.11, R65.20, A41.9 |
