# Supplementary material for: First-year results of the Global Influenza Hospital Surveillance Network: 2012–2013 Northern hemisphere influenza season
Source: BMC Public Health. 2014 Jun 5;14:564. doi: 10.1186/1471-2458-14-564 (PMC4057821; doi:10.1186/1471-2458-14-564)
Supplement: Additional file 2 — Supplemental methods. [file 1471-2458-14-564-S2.doc]

# Supplemental methods

## GIHSN laboratory characteristics and procedures

The following laboratories analysed the samples: Molecular Virology Laboratory of FISABIO – Valencia (Spain); WHO National Influenza Centre, Centre National de Référence du Virus Influenza Région Sud (France); WHO National Influenza Centre, D.I. Ivanovsky Research Institute of Virology of the Ministry of Health of the Russian Federation, Moscow (Russian Federation); WHO National Influenza Centre, Research Institute of Influenza from the Ministry of Health, St. Petersburg (Russian Federation); and the National Influenza Reference Laboratory, Istanbul Faculty of Medicine (Turkey). All of these are National Influenza Centres except for the Molecular Virology Laboratory - FISABIO in Valencia [1].

### Molecular Virology Laboratory of FISABIO, Valencia, Spain

Respiratory viruses were detected using four different screening multiplex real-time PCR assays as described below. Reactions were performed in a Lightcycler 480II apparatus (Roche), using 5 ml of the eluted nucleic acid for each assay.

- Multiplex 1 detected influenza virus type A and influenza virus type B using probes for the Matrix protein [2,3].
- Multiplex 2 detected human coronavirures 229E, NL63, OC43, and HKU1 by using probes from the 1b gene [3]; human metapneuoviruses A and B using probes for the N gene [4]; and human bocavirus using probes for the NP1 gene [5].
- Multiplex 3 detected parainfluenza viruses 1, 2, 3, and 4 using probes for the HN gene [6]; adenovirus using probes for the Hexon gene [6]; and respiratory syncytial viruses (RSV) A and B using probes for the NC gene [6].
- Multiplex 4 detected human rhinovirus using probes for the 5’-untranslated region [7]; and the human ribonucleoprotein P gene, included as internal control for the quality control of the sample, extraction and amplification.

Results for viruses were considered negative only if amplification of the human ribonucleoprotein P gene was positive. Laboratory procedures to prevent PCR contamination were strictly followed and positive (purified viral nucleic acids from Vircell) and negative controls (without sample and/or nucleic acid) were included.

For influenza A-positive samples, RT-PCR was performed as described previously [8] for the HA gene for virus typing (influenza A H1N1pdm09, previous season H1N1, and H3N2) and for the matrix gene as confirmation. For influenza B-positive samples, RT-PCR was performed as describe previously [9] for the HA gene to distinguish B/Yamagata and B/Victoria lineages.

### Centre national de Référence du Virus Influenza Région Sud (France)

The procedures in France were in accordance with the Terms of Reference of the National Influenza Centre [10]. Details of the techniques used for virus detection and characterization are described elsewhere [11-14].

### D.I. Ivanovsky Research Institute of Virology (Moscow, Russian Federation)

Real-time PCR was performed using a Ribo-prep kit (Ecoli) to extract RNA and DNA, a [Reverta-L](http://www.interlabservice.ru/en/catalog/index.php?sid=1037&id=4828) kit (Ecoli) for reverse transcription, an influenza virus A real-time PCR kit (AmpliSens) to amplify A/H1N1 and H3N2 influenza genes, and an in-house kit for influenza B lineages. Reactions were performed in Rotor Gene 6000 (Corbett Research, Australia) and DTlite Real-Time PCR System (DNA technology, Russia).

### Federal and National Influenza Centre in the Research Institute of Influenza (St. Petersburg)

RNA was isolated from 150-200 μL of Viral Transfer Medium containing 2 nasal swabs using an AmpliSense RIBO-prep kit or Qiagen RNeasy Mini kit. RT-PCR for influenza A and B viruses was performed using an AmpliSense Influenza virus A/B-FL kit (InterLabService) with reverse transcription with a Reverta-L kit (InterLabService) or using a OneStep RT-PCR Kit (Qiagen) with CDC primers and probes.

Yamagata or Victoria lineage influenza B virus specific sequences were determined in all influenza B virus-positive specimens using Qiagen OneStep RT-PCR Kit with WHO-recommended primers and probes. Real-time PCR was carried out on Rotor-Gene 6000 (Corbett Research) or CFX96 Touch Real-Time PCR Detection System (BIO-RAD).

Virus isolation was performed in MDCK cell culture and 10-day-old chicken embryos exposed for 72 h at 34ºC according to the approved method [15]. HI test was performed according to the standard method recommended by the WHO with 0.75% suspension of human red blood cell, group 0, Rh+ for influenza A(H3N2) and B viruses. In the case of influenza A/H1N1pdm09 isolates, 1% suspension of chicken erythrocytes was used [16].

Amplification of cDNA was performed by standard method using the original primers. Sequencing of influenza virus A genome fragments (genes HA, NA, M, and NS) was carried out on ABI PRISM 3100-Avant Genetic Analyzer (Applied Biosystems) with a BigDye Terminator Cycle Sequencing Kit v3.1 (Life Technologies).

### National Influenza Reference Laboratory, Turkey

Nasal or nasopharyngeal swabs were collected using Virocult (Medical Wire & Equipment). RNA was extracted using a High Pure Viral Nucleic Acid Kit (Roche) according to the manufacturer’s instructions. Real-time RT-PCR detection of influenza viruses was performed using an ABI 7500 platform with CDC primers and probes according to the CDC protocol [17]. Lineage of influenza B isolates was performed using a hemagglutination inhibition assay with reference viruses and related ferret antisera provided by the WHO collaborating centre in London, UK.

### References

[1] WHO. Influenza. Global Influenza Surveillance and Response System (GISRS) and laboratories(2013) National Influenza Centres. Available at: http://www.who.int/influenza/gisrs_laboratory/national_influenza_centres/list/en/index3.html. Accessed: January 16, 2014.

[2] He J, Bose ME, Beck ET, Fan J, Tiwari S, et al.(2009) Rapid multiplex reverse transcription-PCR typing of influenza A and B virus, and subtyping of influenza A virus into H1, 2, 3, 5, 7, 9, N1 (human), N1 (animal), N2, and N7, including typing of novel swine origin influenza A (H1N1) virus, during the 2009 outbreak in Milwaukee, Wisconsin. J Clin Microbiol 47: 2772-2778.

[3] Suwannakarn K, Payungporn S, Chieochansin T, Samransamruajkit R, Amonsin A, et al. (2008) Typing (A/B) and subtyping (H1/H3/H5) of influenza A viruses by multiplex real-time RT-PCR assays. J Virol Methods 152: 25-31.

[4] Kuypers J, Martin ET, Heugel J, Wright N, Morrow R, Englund JA. (2007) Clinical disease in children associated with newly described coronavirus subtypes. Pediatrics 119: e70-e76.

[5] Matsuzaki Y, Takashita E, Okamoto M, Mizuta K, Itagaki T, et al. (2009) Evaluation of a new rapid antigen test using immunochromatography for detection of human metapneumovirus in comparison with real-time PCR assay. J ClinMicrobiol 47: 2981-2984.

[6] Neske F, Blessing K, Tollmann F, Schubert J, Rethwilm A, et al. (2007) Real-time PCR for diagnosis of human bocavirus infections and phylogenetic analysis. J ClinMicrobiol 45: 2116-2122.

[7] van de Pol AC, van Loon AM, Wolfs TF, Jansen NJ, Nijhuis M, et al. (2007) Increased detection of respiratory syncytial virus, influenza viruses, parainfluenza viruses, and adenoviruses with real-time PCR in samples from patients with respiratory symptoms. J ClinMicrobiol 45: 2260-2262.

[8] WHO(2012) Real-time PCR group protocol #2, WHO molecular diagnosis of influenza virus in humans, November 2012 update.. Available at: http://www.who.int/influenza/gisrs_laboratory/molecular_diagnosis_influenza_virus_humans_update_201211.pdf. Accessed: January 16, 2014.

[9] WHO(2011) Real-time PCR group protocol #1, WHO molecular diagnosis of influenza virus in humans. Available at: http://www.who.int/influenza/resources/documents/molecular_diagnosis_influenza_virus_humans_update_201108.pdf. Accessed: January 16, 2014.

[10] WHO Global Influenza Programme. Terms of reference for National Influenza Centres. Available at: http://www.who.int/influenza/gisn_laboratory/national_influenza_centres/terms_of_reference_for_national_influenza_centres.pdf. Accessed: January 16, 2014.

[11] Lina B, Valette M, Foray S, Luciani J, Stagnara J, et al. (1996) Surveillance of community-acquired viral infections due to respiratory viruses in Rhone-Alpes (France) during winter 1994 to 1995. J ClinMicrobiol 34: 3007-3011.

[12] Casalegno JS, Ottmann M, Duchamp MB, Escuret V, Billaud G, et al. (2010) Rhinoviruses delayed the circulation of the pandemic influenza A (H1N1) 2009 virus in France. Clin Microbiol Infect 16: 326-329.

[13] Escuret V, Cornu C, Boutitie F, Enouf V, Mosnier A, et al. (2012) Oseltamivir-zanamivirbitherapy compared to oseltamivir monotherapy in the treatment of pandemic 2009 influenza A(H1N1) virus infections. Antiviral Res 96: 130-137.

[14] WHO CDC (2009) Protocol of realtime RTPCR for swine influenza A(H1N1); CDC REF. #I-007-05; version 2009:Swine Influenza. Available at: http://www.who.int/csr/resources/publications/swineflu/CDCrealtimeRTPCRprotocol_20090428.pdf. Accessed: January 14, 2014.

[15] Somina A, Burtseva E, Eropkin M, Karpova L, Zarubaev V, et al. (2013) Influenza surveillance in Russia based on epidemiological and laboratory data for the period from 2005 to 2012. Am J Infect Dis 9: 77-93.

[16] WHO (2011) Global Influenza Surveillance Network, Manual for the laboratory diagnosis and virological surveillance of influenza. Available at: http://whqlibdoc.who.int/publications/2011/9789241548090_eng.pdf. Accessed: January 16, 2014.

[17] CDC (2009) CDC protocol of realtime RTPCR for swine influenza A(H1N1). Available at:<http://www.who.int/csr/resources/publications/swineflu/CDCrealtimeRTPCRprotocol_20090428.pdf>. Accessed: January 23. 2014.
