# Supplementary material for: First-year results of the Global Influenza Hospital Surveillance Network: 2012–2013 Northern hemisphere influenza season
Source: BMC Public Health. 2014 Jun 5;14:564. doi: 10.1186/1471-2458-14-564 (PMC4057821; doi:10.1186/1471-2458-14-564)
Supplement: Additional file 3: Figure S1 — Length of hospital stay by coordinating site and influenza strain. Boxes indicate interquartile ranges, bars indicate the upper and lower adjacent values, and points indicate outliers. [file 1471-2458-14-564-S3.doc]

**Supplemental Figure 1. Length of hospital stay by coordinating site and influenza strain.** Boxes indicate interquartile ranges, bars indicate the upper and lower adjacent values, and points indicate outliers.
